# Endothelial glycocalyx glycan expression in lymphatic vasculature in mice

**DOI:** 10.3389/fcell.2026.1910204

**Published:** 2026-08-26

**Authors:** Issahy Cano, Esak Lee

**Affiliations:** 1 Department of Biomedical and Translational Sciences, College of Veterinary Medicine, Cornell University, Ithaca, NY, United States; 2 Nancy E. and Peter C. Meinig School of Biomedical Engineering, Cornell University, Ithaca, NY, United States

**Keywords:** carnoy’s fixative, chondroitin sulfate, heparan sulfate, initial lymphatics, lymphatic glycocalyx, lymphatic vessel-on-chip, LYVE-1, microfluidic device

## Abstract

Lymphatic vessels are crucial for the drainage and transport of interstitial fluids, cells, and macromolecules. Like all other cells, lymphatic endothelial cells express a cell-surface glycocalyx, composed of carbohydrate-enriched molecules, that contributes to endothelial barrier maintenance and cell-cell communication. Although the endothelial glycocalyx has been increasingly studied in blood endothelium, little has been done to characterize it in lymphatics. In this study, among a variety of tested fixatives, Carnoy’s fixative, a solution of ethanol, chloroform, and acetic acid, was determined to best preserve endothelial glycocalyx epitopes in lymphatics-on-chip microfluidic devices and mouse tissue sections. Dermal, lung, and lymph node tissues were isolated from male C57BL/6J mice and fixed with Carnoy’s fixative. We aimed to determine the presence of prominent glycocalyx components, including heparan sulfate and chondroitin sulfate, across different mouse organs to characterize organ-specific differences. We hypothesized that, due to the different organ environment demands for fluid clearance and immune surveillance, the expression of major glycocalyx components would be heterogeneous. However, immunofluorescence imaging of these tissues demonstrated the presence of these glycans within LYVE-1-positive structures across the tested tissues. This conclusion provides a basis for further characterization and understanding of the composition, function, and potential alterations of the lymphatic endothelial glycocalyx across very distinct organs/tissues and conditions.

## 1 Introduction

The cell surface glycocalyx (GCX) is found on all cells and comprises a dynamic mesh of carbohydrate-rich molecules, including glycosaminoglycans and glycoproteins. Endothelial cells line vessel walls in a tight, organized manner, leading to a unique GCX expression pattern that results in an emergent layer at the luminal cell surface. This endothelial GCX (eGCX) surface layer serves as an interface between the endothelial wall and luminal contents, playing major roles in barrier maintenance, cell signaling, and immune trafficking (Zhengping Hu et al., 2021). Prominent components of the GCX, such as heparan sulfate (HS) and chondroitin sulfate (CS), are made up of carbohydrate chains that present an overall negative charge, contributing an electrostatic force component to the barrier, repelling anionic macromolecules like proteins such as albumin and attracting cationic ones (Alphonsus and Rodseth, 2014; Hayes et al., 2008; Nan et al., 2023; Ueda et al., 2004). The thickness of the eGCX impacts trans-vascular fluid dynamics. At smaller thicknesses, the eGCX allows for more facilitated transfer of luminal and interstitial fluid when compared to a more robustly expressed eGCX (Smilowitz et al., 2021; Suzuki et al., 2022). Inflammatory conditions have been shown to reduce eGCX thickness and produce leakier vessels (Suzuki et al., 2022; Arokiasamy et al., 2019; Gao and Lipowsky, 2010). Furthermore, due to the mechano-transduction role of the glycocalyx, flow conditions within vessels also influence the expression of the eGCX layer. Endothelial cells exposed to flow conditions will express a thicker, more polarized glycocalyx near cell junctions (Zeng and Tarbell, 2014). HS is the most abundant glycosylation found across the GCX, comprising about 50%–90% of the glycoconjugates that constitute the layer (Florian et al., 2003). Previous studies have shown that within the blood eGCX, HS and CS glycans are expressed in a 4:1 ratio, respectively; however, this has not been confirmed in the lymphatic eGCX (Rapraeger et al., 1985). Protein cores are glycosylated with HS during post-translational modifications within the Golgi apparatus and are trafficked to the cell surface (Leisico et al., 2022). Proteins such as syndecans, glypicans, and mucins are heavily glycosylated with HS glycans, which contribute to their roles at the cell surface (Bernfield et al., 1999). Additionally, many cell-surface receptors and growth factor ligands, such as vascular endothelial growth factor (VEGF), have HS-binding domains that mediate interactions within the GCX layer. One such impact is the sequestration of ligands by the GCX HS, thereby regulating endothelial signaling pathways (Nagarajan et al., 2018; Ricard-Blum and Lisacek, 2017). The eGCX also provides a physical shield that restricts interactions between luminal contents and the endothelial wall. The activity of cell-surface adhesion molecules, such as intercellular adhesion molecule 1 (ICAM-1) and E-selectin (CD62E), is physically suppressed by a thick GCX. However, once the eGCX is degraded, cell-surface adhesion molecules become exposed and available for close interaction with circulating immune cells, facilitating cell rolling and adhesion. Maintaining a balanced expression of the eGCX is vital for lymphatic function.

Despite its important role in maintaining endothelial function, the eGCX is relatively understudied due to its fragile structure and limited expression outside the *in vivo* environment. Standard fixation methods often dehydrate or destroy the eGCX layer, making it difficult to preserve and study. Standard fixatives such as paraformaldehyde (PFA) are great for preserving certain types of molecules, such as proteins, through crosslinking, which does not target carbohydrates. Reagents such as acetone, ethanol, and formalin are effective for rapid preservation of cell and tissue morphology; however, they may cause dehydration or fail to fully stabilize the eGCX layer. The addition of chloroform or glacial acetic acid is intended to prevent shrinkage caused by the primary fixative. Carnoy’s fixative is a solution of 60% ethanol, 30% chloroform, and 10% glacial acetic acid (Carnoy 1884). The ethanol helps fix cellular molecules through dehydration, while the acetic acid and chloroform help maintain molecular structure and size. The combination of these, in solution, shows promise for rapid preservation of the eGCX layer while mitigating harm to it. Furthermore, standard two-dimensional cell culture models fail to yield a robust eGCX. Under these conditions, a lack of shear stress and three-dimensional structure results in a thinner and more discontinuous eGCX that fails to adequately represent the layer (Chappell et al., 2009). Three-dimensional microfluidic devices provide the necessary structure and flow conditions for proper expression of the eGCX.

Lymphatic vasculature can be found amongst most tissues, where it plays vital roles in immune trafficking and fluid clearance. Differentiated endothelial cells make up the lymphatic endothelial wall and differ from blood endothelial cells predominantly by their expression of Prospero Homeobox 1 (PROX-1), a master driver of lymphatic expression; Lymphatic vascular endothelial hyaluronan receptor-1 (LYVE-1), podoplanin, and VEGF receptor 3 (Zhaoliang Hu et al., 2024; Hu et al., 2019). Lymphatics are composed of two major vessel subtypes, initial and collecting lymphatics, as well as specialized lymphatic structures. Initial lymphatics are blunt-ended capillary vessels found throughout peripheral tissues and serve as the primary exchange points between the interstitium and the lymphatic network, where button-like junctions between lymphatic endothelial cells permit the uptake of fluid, cells, and macromolecules. Initial lymphatics lead to intermediate pre-collecting lymphatics and finally larger collecting lymphatics, which are valved to restrict backflow. Eventually, lymph flows into the bloodstream at the junction of the lymphatic and subclavian veins. Together, this network of vessels provides a means to maintain homeostatic conditions as fluids are accumulated from blood flow. Lymphatic vessels demonstrate a slower flow rate than blood vessels, with an average of 1–2 dyne per cm^2^ and a peak of around 12 dyne per cm^2^ (Angeli and Lim, 2023). As lymph travels through the lymphatic vasculature, it encounters larger lymphatic structures, such as lymph nodes. Lymph nodes are secondary lymphoid organs composed of lymphatic endothelium, stromal cells, and adaptive immune cell populations. Slow-flowing lymph enters lymph nodes through afferent lymphatic vessels and is filtered within immune cell niches, enabling antigen presentation and the activation of B and T cell populations.

Body organs fulfill distinct roles with different immune and fluid clearance demands. Organs such as the lungs and dermis house a large number of lymphatic vessels to allow efficient fluid clearance and, due to their constant exposure to the external environment, greater immune surveillance. On the other hand, tissues such as the brain and cornea lack lymphatic vasculature. Additionally, the limited eGCX research almost exclusively focuses on the blood endothelium, leaving much to be desired in understanding the lymphatic eGCX. Studies have begun to characterize the thickness of the lymphatic eGCX, with a predominant focus on collecting lymphatics (Gianesini et al., 2023). However, the molecular makeup of the lymphatic eGCX has not been fully explored. Furthermore, traditional fixation and collection methods often make it difficult to preserve the endothelial GCX, hindering substantive studies. In this study, we aimed to identify an optimal fixative to preserve and observe the GCX layer and its components among a variety of common fixatives. Additionally, this study aims to provide a preliminary assessment of the organ-specific expression of predominant glycocalyx components. We hypothesized that, due to differences in tissue environments across organs, the emergent expression of eGCX will vary between HS and CS.

## 2 Methods

### 2.1 Lymphatics-on-chip device manufacturing for immunofluorescence imaging

**FIGURE 1:**
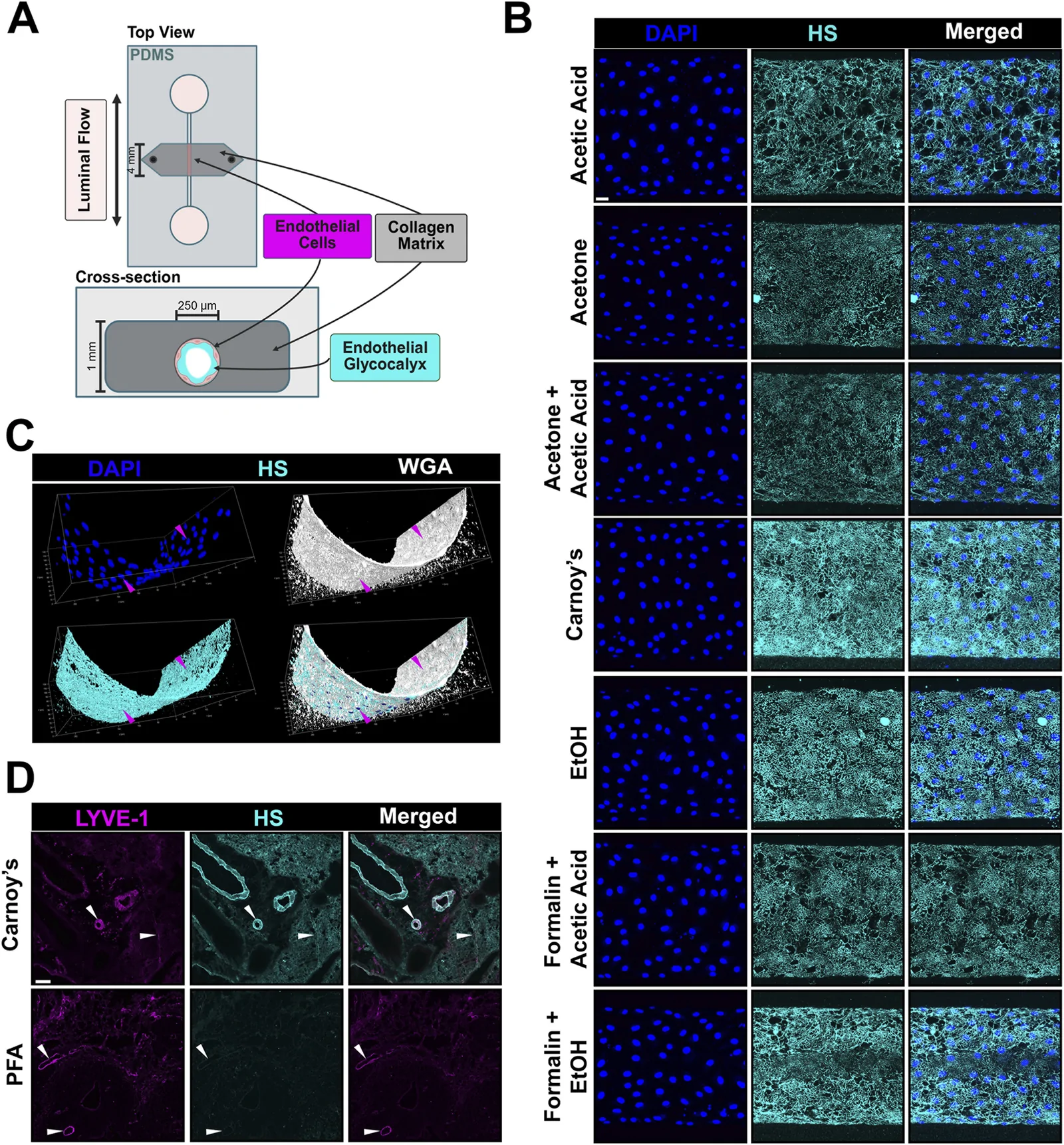
Carnoy’s is an effective fixative for GCX epitopes. **(A)** A schematic illustration of the lymphatic vessel‐on‐chip device detailing the different components housing the lymphatic endothelial cells. **(B)** Engineered lymphatic vessels were cultured within microfluidic devices until they expressed a eGCX. Different fixative solutions were used to fix the cells. Z‐stack images taken with confocal microscopy (40×) were processed by producing a maximum projection and assessed for their effectiveness in preserving HS, which was visualized using immunofluorescence. Carnoy’s fixative best preserved the eGCX layer. Scale bar = 100 µm. **(C)** Confocal Z stack images (40×) of half an engineered vessel within the lymphatic vessel‐on‐chip device were rendered as a 3D image. The glycocalyx was displayed using WGA and HS staining and cells were delineated with DAPI staining. The 3D image is positioned to show both basal and apical (luminal) sides of the vessel. Arrows point to cell nuclei. 3D scale is shown within the image. **(D)** 20 µm mouse lung tissue sections were imaged using immunofluorescence. Confocal Z-stack images (10×) were processed by maximum projection. LYVE‐1 marked lymphatic vasculature. Carnoy’s fixative was compared against PFA fixation. Carnoy’s fixative better preserved HS epitopes as seen using immunofluorescence. Scale bars = 100 µm.

Microfluidic devices were prepared as previously described (Henderson et al., 2021). Device fabrication consisted of casting polydimethylsiloxane (PDMS; Sylgard 184, Dow Chemical Company) on molds, patterning with soft lithography, and cutting to form channels, media reservoirs, and an extracellular matrix central chamber. Devices are attached to a glass coverslip to create an enclosed system. The PDMS surfaces of the device were modified by plasma etching (PE-25 Plasma Cleaner; Plasma, Etch Inc., Carson City, NV) and treating the device with 0.1% poly-L-lysine (#P8920, Sigma) and glutaraldehyde (#16310, Electron Microscopy Sciences) to enable collagen bonding to the PDMS. Inserting 250 µm-diameter needles (#Hs-Gp, Lhasa Oms) into the channels and filling the ECM chamber with collagen I gel (2.5 mg/mL; #354236, Corning) created the 3D matrix scaffolding for the engineered lymphatic vessel after the needles were removed. Human dermal-derived primary lymphatic endothelial cells (provided by Dr. Young K. Hong, Beth Israel Deaconess Medical Center) were seeded into the channel to form engineered vessels after adhering to the collagen matrix. Cells were cultured in EBM-2 cell culture media (#CC-3156; Lonza) supplemented with the EGM -2 Microvascular Endothelial Cell Growth Medium-2 BulletKit (#CC-3162, Lonza). Devices were cultured on a rocker to induce shear stress at 37 °C, 95% humidity, and 5% CO2 for 7 days to optimize eGCX expression (Figure 1A). Various fixatives were used to fix the cells within the chips overnight at 4 °C. Fixatives include glacial acetic acid (1%; #338826, Sigma), acetone, Carnoy’s fixative, ethanol (EtOH; #T038181000, Thermo Scientific), and Formalin (5%; #HT501128, Sigma) in various combinations. The engineered lymphatic vessels were blocked in 3% bovine serum albumin (BSA; #97061–422, VWR) and then incubated with antibodies against HS (1:200; #370255, Amsbio) or CS (1:200; #C8035, MilliporeSigma) in the blocking buffer overnight at 4 °C. After phosphate-buffered saline (PBS; #45001–130, Corning) washes, devices were incubated with goat anti-mouse IgM Alexa Fluor™ 488 (1:1,000; #A-21042, Life Technologies) and DAPI solution (#62248, Thermofisher). Wheat germ agglutinin (WGA, 1:100; # 25559, AAT Bioquest). Finally, the devices were washed in PBS prior to imaging with an SP8 confocal microscope (Leica, Wetzlar, Germany) with a ×40 objective. Maximum projections of tissue Z-stack images were processed using the LAS X SP8 Control Software (Leica, Wetzlar, Germany).

### 2.2 Immunofluorescence imaging of mouse ear wholemounts

Male mouse ears were collected and fixed in Carnoy’s fixative, a solution of 60% ethanol, 30% chloroform (#97064–678, VWR), and 10% glacial acetic acid, overnight at 4 °C. Each ear was then split into anterior and posterior halves, cleaned of excess collagen and fatty tissue, and blocked in a solution of 5% bovine serum albumin, 10% donkey serum (S30, MilliporeSigma), and 0.3% Triton™ X-100 (#X100, MilliporeSigma) in PBS for one hour at room temperature. Ear tissues were then incubated with antibodies against HS (1:200), CS (1:200), and LYVE-1 (1:100; #67538S, Cell Signaling) in the blocking buffer overnight at 4 °C. After PBS washes, the ears were incubated with goat anti-mouse IgM Alexa Fluor™ 488 (1:1,000) and goat anti-rabbit IgG Alexa Fluor™ 568 (1:1,000; #A11036, Life Technologies) secondary antibodies. Finally, the ear tissues were washed in PBS prior to mounting the ears on microscope slides using ProLong™ Gold Antifade Mountant (#P36934, Invitrogen). Slides were imaged using an SP8 confocal microscope with ×10 and ×40 objectives. Maximum projections of tissue Z-stack images were processed using the LAS X SP8 Control Software (Leica, Wetzlar, Germany).

### 2.3 Immunofluorescence imaging of mouse lung and lymph node tissue sections

For mouse studies, male C57BL/6J mice (The Jackson Laboratory) were used at 6–8 weeks old. Tissues were flash frozen and embedded in O.C.T. Compound (#4583, Tissue-Tek®) immediately after collection. Tissues were then sectioned into 20 µm or 80 µm slices and collected on charged microscope slides (#16004–406, VWR). Tissue sections were thawed and blocked in a solution of 5% BSA, 10% donkey serum, and 0.3% Triton™ X-100 in PBS for one hour at room temperature. Primary antibodies were applied in the blocking solution. Tissues were stained for HS (1:200), CS (1:200),LYVE-1 (1:100), VEGFR3 (1:100; #AF743, R&D Systems), and isolectin B4 (1:400; #I21412, Thermofisher). Secondary antibodies, including goat anti-mouse IgM Alexa Fluor™ 488 (1:1,000) and goat anti-rabbit IgG Alexa fluor™ 568 (1:1,000), were applied to the tissue sections in the blocking solution after PBS washes. After further washes in PBS, tissues were then fixed with Carnoy’s fixative or 1% PFA (#15710, Electron Microscopy Science) for 10 min at −20 °C. Tissue slides were then mounted with ProLong™ Gold Antifade Mountant. Slides were imaged using an SP8 confocal microscope with ×10 and ×40 objectives. Maximum projections of tissue Z-stack images were processed using the LAS X SP8 Control Software (Leica, Wetzlar, Germany).

## 3 Results

### 3.1 Carnoy’s fixative is more effective than PFA at preserving tissue glycans

The defining class of molecules in GCX components is carbohydrates. This results in limitations for traditional fixatives such as PFA, which excel at targeting proteins. Engineered lymphatic vessels within microfluidic devices were cultured to effectively express the eGCX and then subjected to one of a variety of different fixative solutions to determine an optimal fixative (Figure 1B). Among the tested fixatives, those containing ethanol better preserved HS expressed by the lymphatic endothelial cells, with Carnoy’s fixative showing the best results. Carnoy’s fixative, a solution of ethanol, chloroform, and acetic acid, showed greater potential for preserving non-protein molecules found throughout cells. To verify this in tissues, we prepared mouse lung tissue slices, fixed them with either Carnoy’s fixative or 1% PFA, and stained them with antibodies against HS targeting the 10E4 epitope (Figure 1B). The engineered lymphatic vessels within the device were shown to effectively express eGCX, including HS, at the apical layer. A more complete GCX layer was identified using wheat germ agglutinin (WGA), a lectin that binds O-glycans, which are prominent carbohydrate chains common amongst the GCX layer (Figure 1C). While the whole GCX and HS were readily detected using this device, further optimization is necessary to observe other GCX components, such as CS, within the device (Supplementary Figure S1). Strikingly, there is a clear difference in the quality of stained HS across the tissue, depending solely on the fixative used. Carnoy’s fixative was superior in preserving expressed HS glycans in fixed cell and tissue section samples for immunofluorescence applications and was employed for all the following sample fixation (Figure 1D).

### 3.2 Mouse dermal lymphatic vessels exhibit dynamic expression of HS and CS

**FIGURE 2:**
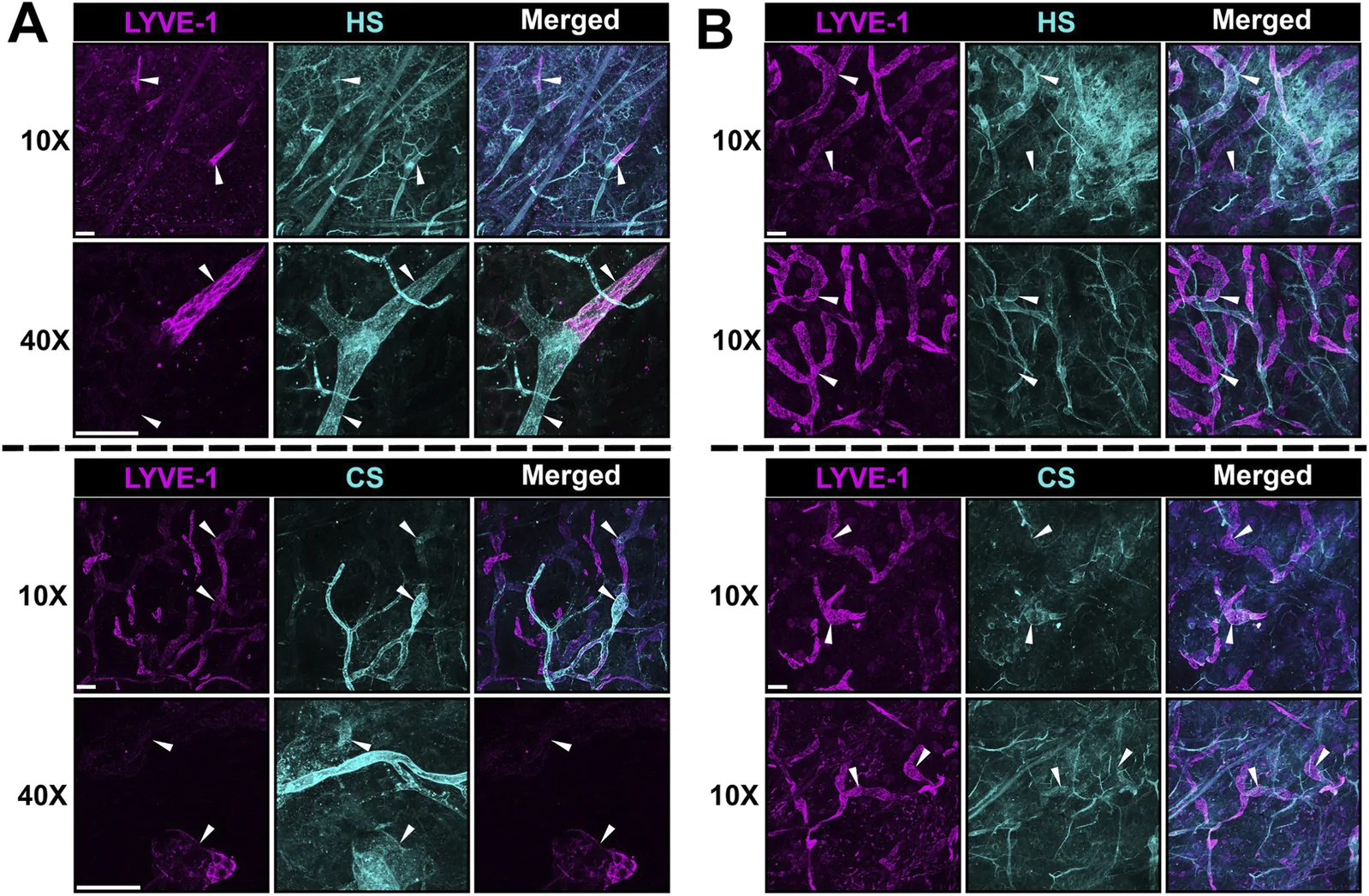
Dermal lymphatics co-express HS and CS in mice. **(A)** Mouse ear wholemounts were prepared for immunofluorescence imaging using Carnoy’s fixative and imaged by confocal microscopy (10× and 40×). 2D maximum projections are shown. Both HS and CS glycans are seen amongst LYVE‐1 positive and negative lymphatic vessels. Scale bars = 100 µm. **(B)** Additional fields of view are shown to better represent the heterogenous expression of HS and CS throughout the lymphatic plexus. Images were taken by confocal microscopy (10×) and presented as 2D maximum projections. Scale bars = 100 µm.

Skin tissue is broken down into three distinct layers. The most superficial layer is the epidermis, which lacks lymphatic vasculature, followed by the dermis, which is rich in vascular networks, and finally the hypodermis, which connects the skin to other tissues such as muscle or bone (Chen et al., 2026; Arokiasamy et al., 2019). Dermal lymphatics are vital for immune surveillance and fluid clearance, with superficial initial lymphatics draining to deeper collecting lymphatics. To visualize the dermal lymphatic plexus, we split mouse ears and stained them for prominent GCX components, along with LYVE-1 as a marker for initial lymphatics (Figure 2A). Low-magnification images (10×) revealed a complex network of LYVE-1-positive lymphatics that continue into LYVE-1-negative and valved precollector lymphatics. Fluorescent antibodies against HS stained the glycan throughout the initial and pre-collector lymphatic structures. However, HS localization on LYVE-1-positive lymphatics was variable throughout the plexus. High-magnification (40×) images further demonstrate the expression of both HS and CS within LYVE-1-positive vessels that continue into the pre-collector lymphatics. Further observations across different fields of view of the mouse ear lymphatic plexus revealed heterogeneous expression of HS and CS (Figure 2B). Previous studies have demonstrated rapid, enzyme-driven changes in HS GCX expression in dermal tissues, which are thought to enable dynamic responses to abrupt fluid buildup and inflammatory conditions (Arokiasamy et al., 2019). This suggests a non-centralized change in HS expression across the dermal lymphatic eGCX. A similar expression pattern is observed for CS; however, a mechanism underlying dynamic changes in CS expression in the eGCX has yet to be demonstrated.

### 3.3 Mouse lung tissues robustly express both HS and CS

**FIGURE 3:**
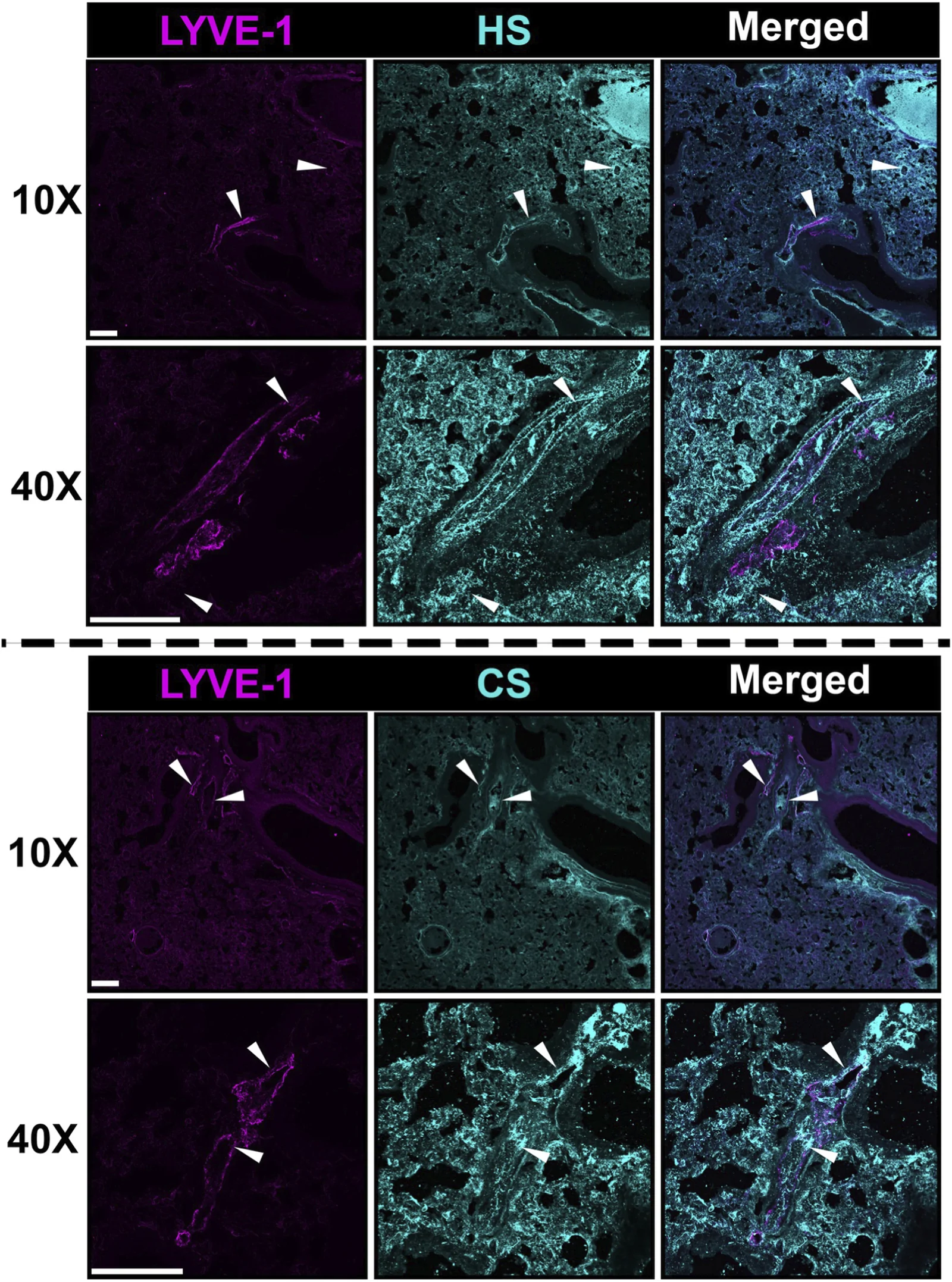
Both HS and CS are prominently expressed in the mouse lung. 20 µm mouse lung tissue sections were fixed using Carnoy’s fixative and imaged using immunofluorescence. Confocal Z‐stack images (10× and 40×) were processed by maximum projection into 2D images. LYVE‐1 marked lymphatic vasculature. HS and CS were observed associated with lymphatic eGCX. Scale bars = 100 µm.

Initial lymphatics are widely distributed throughout the lung parenchyma, facilitating rapid fluid clearance and immune surveillance, as the lung is exposed to the external environment and must efficiently perform gas exchange (Yang and Schmidt, 2013). Lung lymphatics are found in both the interalveolar and intralobular regions, typically within perivascular and subpleural niches (Yang and Schmidt, 2013). Lymphatic vascular, marked with LYVE-1 staining, was observed within the interalveolar region (Figure 3). These vessels showed robust and consistent expression of both HS and CS. This suggests either that, unlike dermal lymphatics, lung lymphatics may lack dynamic lymphatic eGCX remodeling, or the tissue environment within the lung parenchyma is more stable, leading to less degradation of the eGCX layer. Strikingly, within lung tissue, larger non-lymphatic vessels have been shown to express LYVE-1, which may contribute to this observation (Reed et al., 2019). Attempts to stain for lung tissues with an alternative lymphatic marker, VEGFR3, failed to effectively stain thicker (80 µm) lung slices, however counter staining blood vessels using isolectin B4 (IB4) reinforced the finding of LYVE-1 positive blood vasculature (Supplementary Figures S2A and S2B).

Notably, in the lung, endothelial cells are not the only cells with an emergent apical GCX layer. Alveolar epithelial cells similarly express a GCX, which helps protect the vulnerable tissue from the external environment (Haeger et al., 2018; Li et al., 2021). HS and CS have also been shown to be prominent components in this context, with similar protective and cell-signaling regulatory roles (Haeger et al., 2018; LaRivière and Schmidt, 2018; Li et al., 2021). Given the importance of protecting lung tissue, maintaining stable, robust expression of HS and CS across multiple locations within the tissue may be necessary; thus, the eGCX may be less dynamic until injury or infection.

### 3.4 Mouse lymph node lymphatics express both HS and CS

**FIGURE 4:**
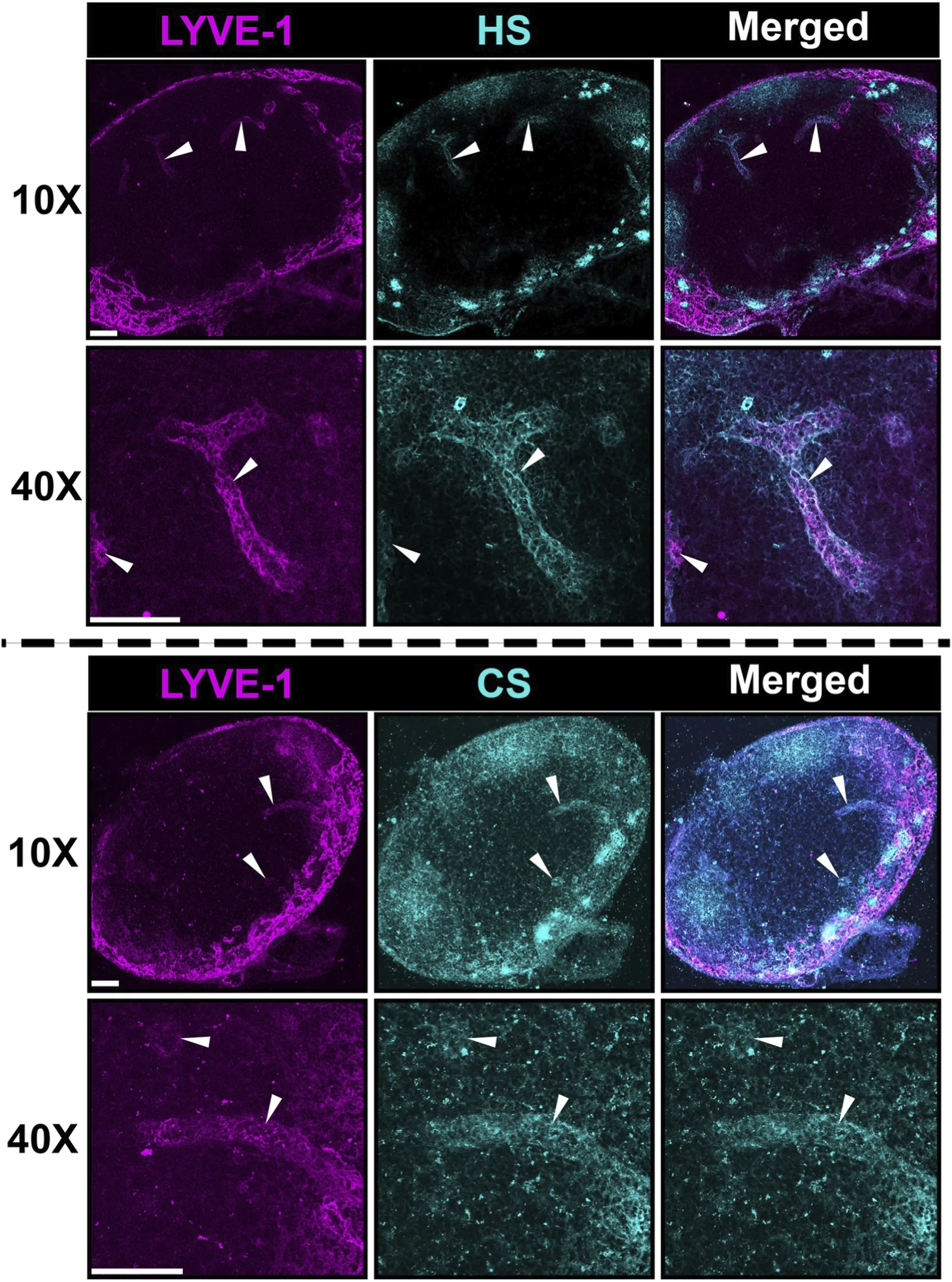
Both HS and CS are present in mouse lymph node lymphatic vessels. 20 µm mouse lymph node tissue sections were fixed using Carnoy’s fixative and imaged using immunofluorescence. Confocal Z‐stack images (10× and 40×) were processed by maximum projection into 2D images. LYVE‐1 marked lymphatic vasculature. Both HS and CS were observed associated with lymphatic vessel eGCX. Scale bars = 100 µm.

Lymphatic endothelium comprises the outer capsule of the lymph node structure, which houses B cell follicles within the cortex, followed by the paracortex, containing the T cell regions. Within the lymph node, blood and lymphatic vessels run throughout the tissue to maintain critical immune regions and provide routes for immune cell trafficking. Staining the lymph node with a lymphatic marker highlighted the lymphatic endothelium that forms the capsular layer, as well as the lymphatic vessels that traverse the intra-lymph node space (Figure 4). Both HS and CS glycans were identified at LYVE-1-positive vascular structures as well as within larger patches in the cortex region. Previous work has shown that both HS and CS play a critical role in B cell maintenance, proliferation, and activation. Within *in vitro* and *in vivo* studies, CS has been shown to be necessary for B cell proliferation and activation, while HS is required for proliferation and proper B cell selection (Brühl et al., 2014; Chen et al., 2023). This contributes to the explanation for the expression of these specific glycans at or near the follicular regions of the cortex. Vascular expression of these GCX components may have similar roles in maintaining proper immune cell function and potentially trafficking.

## 4 Discussion

The eGCX has remained understudied in the lymphatic endothelium, largely because its fragile structure is poorly preserved by conventional fixation. Here we established Carnoy’s fixative as a reliable fixative for retaining GCX glycans for immunofluorescence imaging in both tissue sections and whole mounts, and apply it to define the composition of the lymphatic eGCX *in situ*. Using this approach, we show that HS and CS are both constitutive components of the lymphatic endothelial surface, and that CS is associated with LYVE-1-positive lymphatic vessels, a relationship that has not been previously demonstrated. We further show that this composition is conserved across dermal, pulmonary, and lymph node lymphatics, and is recapitulated in an engineered lymphatic vessel-on-chip model. Together, these results provide both a characterization of the lymphatic eGCX and a practical experimental platform for studying it.

The mammalian GCX was first described more than 50 years ago, and only recently have major strides been made in understanding its role in homeostasis and disease (Martínez-Palomo, 1970). A major roadblock to advancing research in GCX biology has been its fragile structure, making it difficult to preserve. In this study, we demonstrated that for immunohistochemistry applications, Carnoy’s fixative preserves carbohydrate epitopes more effectively than other typical fixatives like PFA in tissue cryosections or formalin in cell culture and that was compatible with whole-mount preparations, as shown in ear dermal stainings. Developing and validating novel methodologies to better handle tissues for studying the GCX is necessary for greater advancement in understanding its underlying mechanisms, which are relevant to both tissue maintenance and pathology. Because this protocol requires no specialized equipment and integrates with routine immunofluorescence workflows, it is readily adoptable. We additionally demonstrated that microfluidic devices housing engineered lymphatic endothelial cells effectively express and visualize components of the eGCX, as it provides optimal conditions recapitulated from tissue biology, but within an accessible platform. Organ-on-chip microfluidics and Carnoy’s fixative are promising approaches for further studying the eGCX in applications not only to lymphatics but also to blood vasculature and other GCX-bearing epithelia. This established system allows for greater possibilities for manipulating GCX conditions, such as enzymatic removal, shear modulation, or inflammatory stimulation, which are not easily isolated *in vivo*.

The bulk of the work characterizing the eGCX has been done in blood endothelium, and much has been left to be confirmed within the context of the lymphatic endothelium. HS has been well established to be present in both endothelia, though its role is understood almost solely from studies on blood endothelium. CS, on the other hand, has mainly been confirmed to be expressed across the blood endothelium (Reitsma et al., 2007). Our demonstration of CS expression on LYVE-1-positive lymphatic capillaries therefore extends a defined structural and functional element of the blood endothelial surface into the initial lymphatic compartment. Notable roles of CS within the blood eGCX include its contributions to the layer’s structure, barrier function, leukocyte rolling, and regulation of cytokine activity (Gao and Lipowsky, 2010; Hirose et al., 2002). Each of these functions has a direct lymphatic counterpart. Initial lymphatics operate as an absorptive rather than a retentive barrier, taking up interstitial fluid and macromolecules through discontinuous button junctions. A charged glycan layer overlying these entry points is positioned to act as a size- and charge-selective filter governing what enters the vessel lumen. Lymphatic capillaries are also the principal entry route for dendritic cells and lymphocytes into the afferent lymph, a process directed by endothelial CCL21 that is itself immobilized and presented by glycosaminoglycans, and occurring at a surface where LYVE-1 engages hyaluronan on the leukocyte. The glycocalyx thus sits at the interface where both fluid uptake and immune cell transmigration are determined, and its composition is a plausible regulator of each. With this in mind, GCX components such as CS may have different roles or expression patterns in this context. However, organ-specific influences on LYVE-1 positive lymphatics did not impact the expression of CS.

Despite substantial differences in the mechanical, immunological, and fluid-handling demands placed on lymphatics in these tissues, the core glycan composition of the endothelial surface is preserved, indicating that it constitutes a baseline feature of the lymphatic endothelium rather than a tissue-adapted one. This shifts the likely focus of functional regulation from organ-specific composition at homeostasis, to instead variations arising at the level of core protein identity, sulfation pattern, or layer thickness, parameters not resolved by the labeling approach used here, or may emerge dynamically under inflammatory or pathological conditions. Our analysis was limited to two glycosaminoglycan classes in healthy tissue and did not include hyaluronan, and characterization of collecting vessels remains to be performed. Establishing a conserved homeostatic baseline is nonetheless a prerequisite for detecting the remodeling that these questions require.

The eGCX has major implications in endothelial function and pathology yet its characteristics in the lymphatic endothelium remain largely undefined. Further progress and study need to be undertaken to explore and define the characteristics of the eGCX. The methodologies presented here make several topics experimentally accessible, such as differences in eGCX expression and function across vessel subtypes, sex-determined differences, and differences in disease states, such as in cancer or lymphedema. By providing insights into lymphatic eGCX expression in healthy mouse tissues and validated methods for its preservation and visualization, this study supplies the reference point and the toolkit for that work.

## Data Availability

The original contributions presented in the study are included in the article/Supplementary Material, further inquiries can be directed to the corresponding author.
