# Supplementary material for: Endothelial glycocalyx glycan expression in lymphatic vasculature in mice
Source: Front Cell Dev Biol. 2026 Aug 26;14:1910204. doi: 10.3389/fcell.2026.1910204 (PMC13553795; doi:10.3389/fcell.2026.1910204)
Supplement: Supplementary file 1 [file DataSheet1.pdf]

Supplementary Materials

Endothelial glycocalyx glycan expression in lymphatic vasculature in mice

Issahy Cano[1], Esak Lee[2*]

[1]Department of Biomedical Sciences, College of Veterinary Medicine, Cornell University, Ithaca, NY, 14853, USA

[2]Nancy E. and Peter C. Meinig School of Biomedical Engineering, Cornell University, Ithaca, NY, 14853, USA

*** Correspondence:**
Esak Lee
el767@cornell.edu

Contents

Supplementary Figure 1. CS was not observed within the current lymphatics-on-chip device.

Supplementary Figure 2. Immunofluorescence imaging of thicker lung sections.

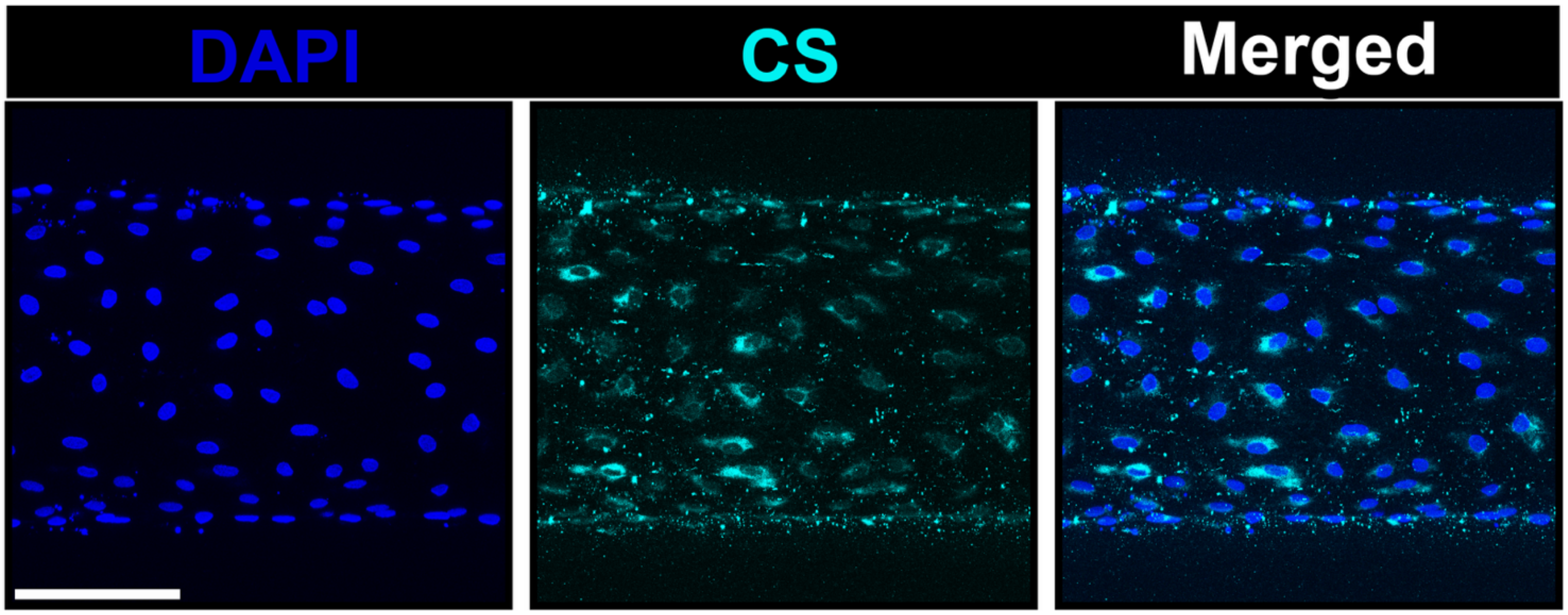

Supplementary Figure 1. CS was not observed within the current lymphatics-on-chip device. Engineered lymphatic vessels were cultured within microfluidic devices and stained for immunofluorescence detection of CS. 2D maximum projections of confocal Z-stacks (40×) are shown. Scale bar = 100 µm.

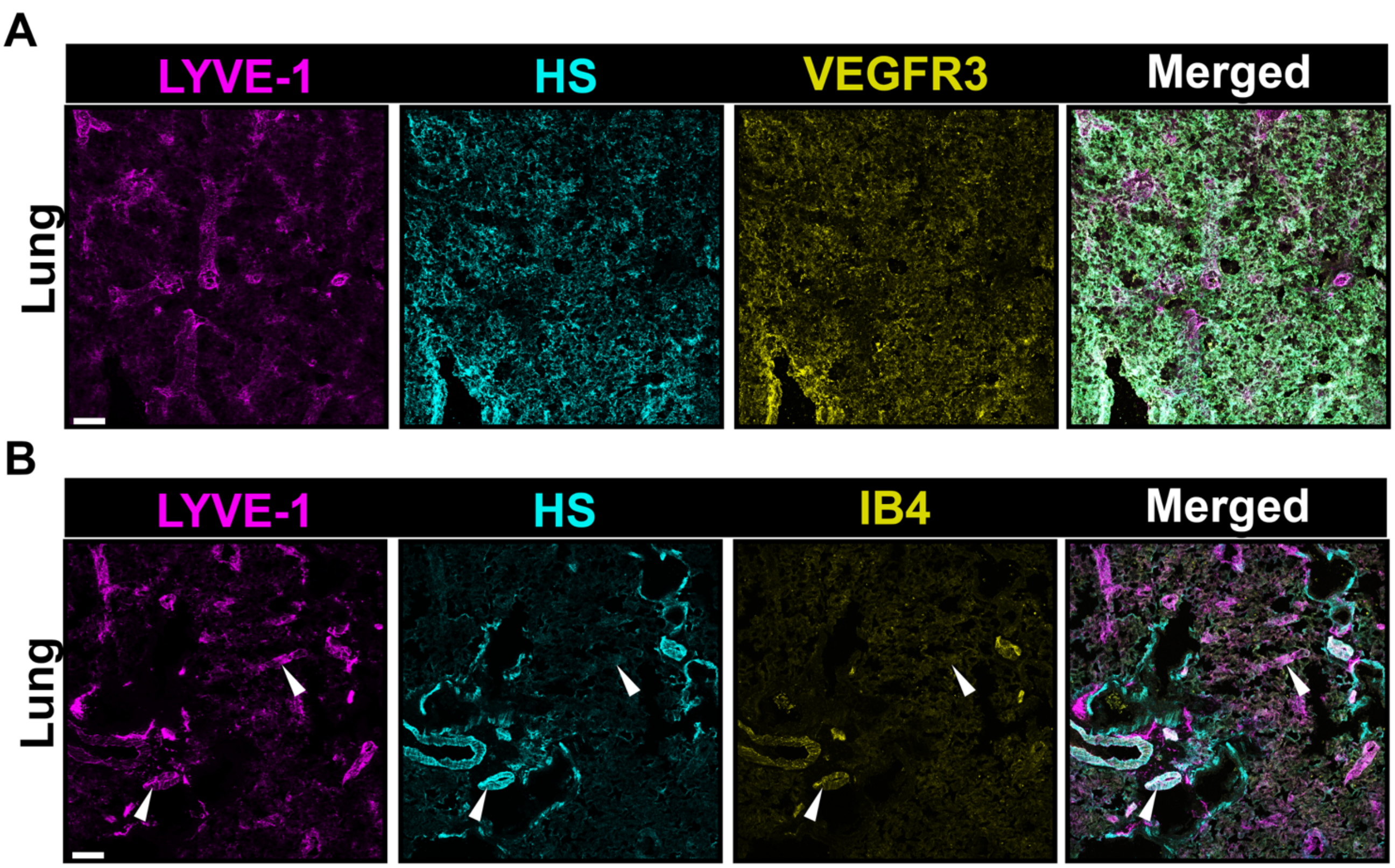

Supplementary Figure 2. Immunofluorescence imaging of thicker lung sections. Embedded lungs were sectioned into 80 µm thick slices. Lung sections underwent antibody staining followed by fixation with Carnoy's fixative. Confocal Z-stack images (10×) are presented as 2D maximum projections. **(A)** An additional lymphatic marker, VEGFR3, is used to discern lymphatic vessels within the lung tissue. **(B)** Blood vessels were counterstained to distinguish between LYVE-1 positive blood vessels and LYVE-1 positive lymphatic vessels. Scale bars = 100 µm.

This is a provisional file, not the final typeset article
